# Examining the causal relationships between interpersonal motivation, engagement, and academic performance among university students

**DOI:** 10.1371/journal.pone.0274229

**Published:** 2022-09-15

**Authors:** Takamichi Ito, Takatoyo Umemoto

**Affiliations:** 1 Faculty of Human-Environment Studies, Kyushu University, Fukuoka, Japan; 2 Faculty of Foreign Studies, Kyoto University of Foreign Studies, Kyoto, Japan; Central China Normal University, CHINA

## Abstract

This study investigates the validity of path models in which interpersonal motivation positively predicts behavioral, emotional, and cognitive engagement, and behavioral engagement positively predicts superior performance in collaborative learning in university classes. The path model was tested using structural equation modeling. The results of the analyses showed that weak positive paths from identified regulation to all three aspects of engagement were substantial. In addition, a weak positive path from intrinsic motivation was significant for emotional engagement. For performance, the weak positive path from behavioral engagement was statistically significant. Based on these results, the implications of the motivation theory and practice in higher education are discussed.

## Introduction

Understanding the autonomous motivation for human interaction in collaborative learning is important for guiding engagement in collaborative activities in the classroom, leading to superior performance. To date, a vast body of research in the educational context has demonstrated that intrinsic motivation is a strong and positive predictor of academic achievement and performance [1–4]. In addition, a number of theoretical and empirical examinations have been accumulated in research on motivation in academic learning, and, as a result, these research findings [5–7] have revealed that intrinsic motivation for learning positively predicts academic engagement and that engagement is a key factor mediating intrinsic motivation and academic performance.

However, previous studies have primarily focused on how intrinsic motivation for individual learning as a personal factor positively influences engagement in academic learning by each person. The classroom is a place for individual learning, as well as for learning with classmates. Interest in and value placed on learning content are important motivational factors, but the kinds of motivations they have for interpersonal relationships in learning situations may also be relevant. To examine the process of academic achievement in collaborative learning in detail, it is necessary to address interpersonal motivation; however, such studies have not been found to any great extent.

### Self-determination theory and interpersonal motivation

Self-determination theory [3, 6] is a major motivational theory that elaborates on intrinsic and extrinsic motivations and clarifies specific functions of multiple motivations. Self-determination theory proposes four types of motivations in order of the degree of self-determination. The first is external regulation, which refers to motivation through rewards, punishment, or coercion from others. The second is introjected regulation, which stems from feelings of shame and anxiety and motivates behavior to maintain self-esteem. The third is identified regulation, in which motivation is based on the perceived value or importance of the behavior. The fourth is intrinsic motivation, which is spontaneous motivation generated by interest, fun, and enjoyment. These different types of motivations are positioned on a continuum of self-determination. In other words, intrinsic motivation has the highest level of self-determination, while external regulation has the lowest.

Previous research [4, 8] based on the self-determination theory has often examined motivation for learning in the academic domain. As research has progressed, studies have been conducted to examine motivation not only in the academic domain, but also in the human relations domain. For example, studies have addressed motivation for prosocial behavior [9] and examined friendship motivation [10, 11]. Motivational factors such as prosocial and friendship motivation have been shown to influence psychological adjustment and individuals’ well-being.

Prosocial and friendship motivation are important aspects of motivation, but considering the collaborative learning situations that are common in classrooms, it may be necessary to look at motivation for group activities. However, from the perspective of the self-determination theory, there has been little research on interpersonal motivation in collaborative activities. Although intrinsic motivation has been considered the strongest predictor of behavior in previous studies, identified regulation has also been shown to play an important role in a variety of contexts [12, 13].

### Motivation, engagement, and educational outcome

Regarding motivation for individual learning, many studies [5, 6] have shown that intrinsic motivation positively determines behavioral, emotional, and cognitive engagement in educational contexts. Walker, Greene, and Mansell [7] have investigated whether intrinsic motivation, identification with academics, and self-efficacy predict cognitive engagement among college students. The results of the path analysis indicated that intrinsic motivation, identification with academics, and self-efficacy positively predicted cognitive engagement. Although the study was conducted on junior high school students, Shih [14] has reported the following results based on self-determination theory: students who learned from personal interest and personal relevance, that is, intrinsic motivation and identified regulation, were more emotionally and behaviorally engaged in schoolwork. Jang [15] has examined the relationship between rationale, motivation, behavioral engagement, and conceptual learning during relatively uninteresting learning activities among undergraduate students; structural equation modeling analysis found significant positive paths from identified regulation to behavioral engagement, and then from behavioral engagement to performance [15]. Furthermore, Saeed and Zyngier [16], through a qualitative case study, have suggested that students with high intrinsic motivation were authentically engaged in their education. As in the previous studies presented above, regarding motivation in individual learning as a personal factor, intrinsic motivation and identified regulation positively determine behavioral, emotional, and cognitive engagement. Similar associations can be assumed in interpersonal motivation of learning situations that require collaboration with others. It is possible that individuals who are motivated by enjoying and valuing interpersonal relationships may be more willing to actively engage in collaborative learning in behavioral, emotional, and cognitive aspects.

Furthermore, previous research has shown that intrinsic motivation and identified regulation lead to a variety of educational outcomes, including academic performance. For example, intrinsic motivation and identified regulation have been found to be positively associated with academic achievement [12], well-being, and school adjustment [13]. In addition, recent studies have shown that identified regulations have more desirable educational outcomes than intrinsic motivation. For example, in a longitudinal study, Otis, Grouzet, and Pelletier [17] found that identified regulation was more strongly positively associated with educational adjustment (i.e., dropout intentions, absenteeism, homework frequency, and educational aspirations) than intrinsic motivation. Koestner and Losier [18] conducted a longitudinal study of academic satisfaction after entering college. The results revealed that intrinsic motivation and identified regulation positively predicted academic satisfaction in the early years, but only identified regulation predicted academic satisfaction in the long run. In addition to these studies, a review of over 40 years of research and a meta-analysis of studies on the undermining effect [2] have shown that intrinsic motivation predicted more unique variance in quality of performance. Although the above studies include findings of individual motivation for learning and activity, it can be assumed that interpersonal motivation would show similar predictions for performance through collaborative learning. Therefore, individuals who are motivated by greater interest and importance in interpersonal relationships will be more willing to engage in academic work with their classmates. This will result in superior performance through collaborative learning.

### Academic engagement and performance

Academic engagement has been demonstrated by several educational studies as a key variable that is a direct and strong predictor of performance [19–21]. Engagement is defined as a student’s active involvement and emotional quality during a learning activity [22]. Engagement has been validated by previous studies in three dimensions: behavioral, emotional, and cognitive [5, 23, 24]. Behavioral engagement includes effort, attention, and persistence while participating in the learning activities [21]. Emotional engagement includes enthusiasm, interest, and enjoyment [21, 25]. Cognitive engagement refers to how students use sophisticated in contrast to superficial learning strategies [26]. Prior research has shown that all three types of engagement are positively associated with academic performance, but path model testing has shown that behavioral engagement, in particular, is a significant positive predictor of academic performance [27–29]. Behavioral engagement is the process of paying attention to a learning task itself. As such, it is assumed to be most directly linked to learning outcomes.

Collaborative learning is a significant educational experience from primary and secondary education to higher education [30–32]. Previous research suggests that engaging in collaborative and cooperative learning can lead to academic achievement [30, 33, 34]. Active engagement in classroom situations, including collaborative learning, will lead to a deeper understanding of the lecture, creative thinking, and better performance in university classes.

### Current study

Based on the findings of previous studies and the presented review, the hypotheses of this study are summarized as follows.

1. Identified regulation and intrinsic motivation will positively predict behavioral, emotional, and cognitive engagement.
2. Behavioral, emotional, and cognitive engagement will positively predict academic performance.
3. Among the three aspects of engagement, behavioral engagement will be the strongest positive predictor of academic performance.

**Fig 1:**
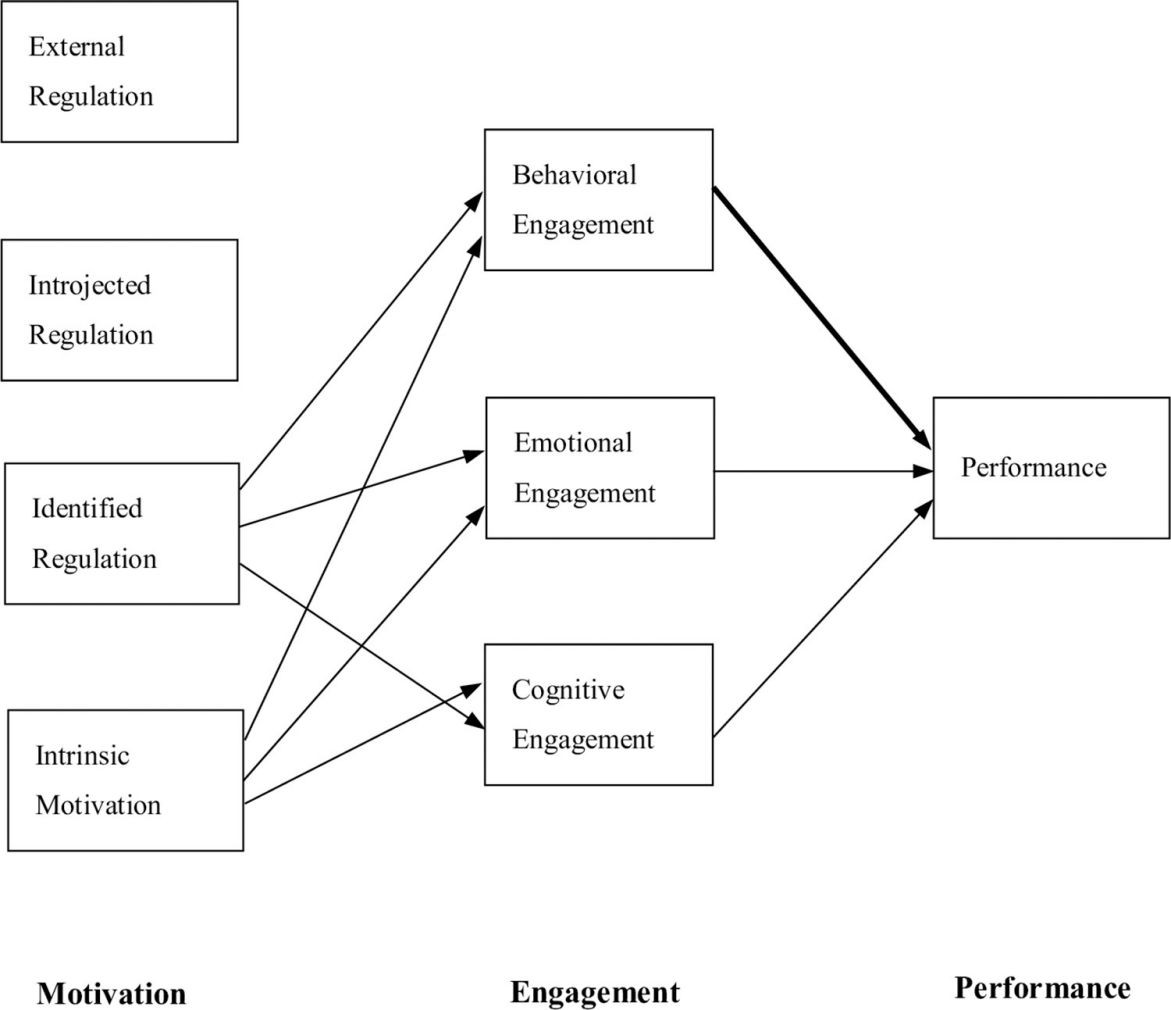
Hypothetical structural equation model for the interpersonal motivation, engagement, and academic performance. Note: The bold arrow indicates the strongest association.

In examining these hypotheses, because engagement is a psychological factor for an individual that is deeply grounded in the actual situation, we will attempt to examine the extent of engagement in university classes that incorporate collaborative learning. In light of previous research [19–21], engagement in the classroom leads to better performance. Performance is measured by the extent to which students show a profound understanding and creative thinking in their essays. Based on the findings of previous studies [5–7], a path model can be postulated in which identified regulation and intrinsic motivation positively predict behavioral, emotional, and cognitive engagement, and behavioral engagement positively predicts superior performance in university classes. This hypothetical structural equation model is illustrated in Fig 1 below. This study empirically investigates the validity of such causal models to obtain suggestions for theory and practice in higher education.

## Method

### Participants

The participants of this study were 363 Japanese undergraduate students (223 male, 124 female, and 16 non-respondents) in their second through fourth years of college. The average participant age was 19.37 (*SD* = 1.26) years old. This study was approved by the research ethics committee of the Faculty of Human-Environment Studies at Kyushu University (approval number 2019–006). The research was conducted according to the guidelines for human subjects and verbal informed consent from the participants was obtained.

### Measures

#### Interpersonal motivation for collaborative learning

To assess interpersonal motivation for collaborative learning, we distributed a questionnaire face-to-face to participants before the lecture. Based on Okada [10], Ryan and Connell [9], and Vallerand and Blssonnette [8], we developed an interpersonal motivation questionnaire to assess the four motivational concepts of external, introjected, identified, and intrinsic reasons for establishing human relationships in collaborative learning. The subscale used to assess each concept was composed of four items; therefore, this motivation scale comprised a total of 16 items. Regarding interpersonal relationships in daily collaborative learning, participants were asked, “Why do you want to be close to or spend time with your group members?”

An example of an external regulation item for this question is “Because I am asked by those around me to build relationships with my members”; of introjected regulation is “Because I feel anxious if I am not close to group members”; of identified regulation is “Because it is important for me to spend time with group members”; and finally, of intrinsic motivation is “Because it is fun to spend time with group members.” Details of the questionnaire items are given in the S1 File. These items were presented on a Likert scale with responses ranging from “1” for “I don’t agree at all” to “5” for “I agree very much.” Scoring was based on responses from 1 to 5, and these scores were used for subsequent analyses. The same is true for all subsequent measurements.

#### Engagement

Immediately after the lecture, including pair work and collaborative learning, we measured behavioral, emotional, and cognitive engagement. Based on Reeve and Tseng [28] and Skinner, Kindermann, Connell, and Wellborn [5], a questionnaire with three aspects of engagement was developed. Behavioral engagement consisted of five items (e.g., “I paid attention in pair work,” “I tried very hard in pair work”). Emotional engagement consisted of five items (e.g., “When we work on something in pair work, I felt interested,” “Pair work was fun”). Cognitive engagement consisted of four items (e.g., “I devised the way to promote learning through pair work,” “I devised the way to deepen the ideas in the discussion through pair work”). Details of the questionnaire items are provided in the S1 Appendix. These items were presented on a 1–7 bipolar response scale, with responses ranging from “1” for “not at all true for me” to “7” for “very true for me.” Scoring was based on responses from 1 to 7, and these scores were used for subsequent analyses.

#### Performance

After the pair work, the students were instructed to think individually and write a report while exercising their creativity and reflecting on the content of the dialogue with their partners. A worksheet (one sheet of A4 size) was distributed, with a column for writing each of the three questions (corresponding to about one-third of the paper space). Based on the essay content, which should be three to five lines long, the students were graded on a scale of A to C. According to the order of rating, the highest grade, A, was scored as three points, while grade C was scored as one point considering the quality and depth of thought. The report was ranked “C” when there was: “lack of explanation,” “mere description of the lecture content as it is,” or “insufficient consideration.” A report that met the following criteria was ranked as “B”: “the explanation of the lecture content includes one’s own ideas,” “original consideration by the author,” and “new information is added to the lecture content.” Rank “A” was scored when: “In addition to one’s own opinion, one’s partner’s ideas are included in the discussion in a valid way,” “One’s own ideas are contrasted with those of one’s partner, and novel ideas are formed,” “Based on the dialogue, a deep discussion is created.”

The first author explained the grading criteria in advance and two graduate students majoring in educational psychology independently graded the reports. Ten reports were randomly selected, and two graders were checked to ensure that they fully understood the grading criteria. Subsequently, another 100 descriptions were randomly selected for further grading, with an inter-rater agreement rate of 76.67%. Reports with inconsistent grades were carefully discussed by the first author and two graders, and the final grades were determined. After confirming that a common understanding of the grading criteria had been obtained, the two raters graded the remaining reports separately.

#### Procedures

**Fig 2:**
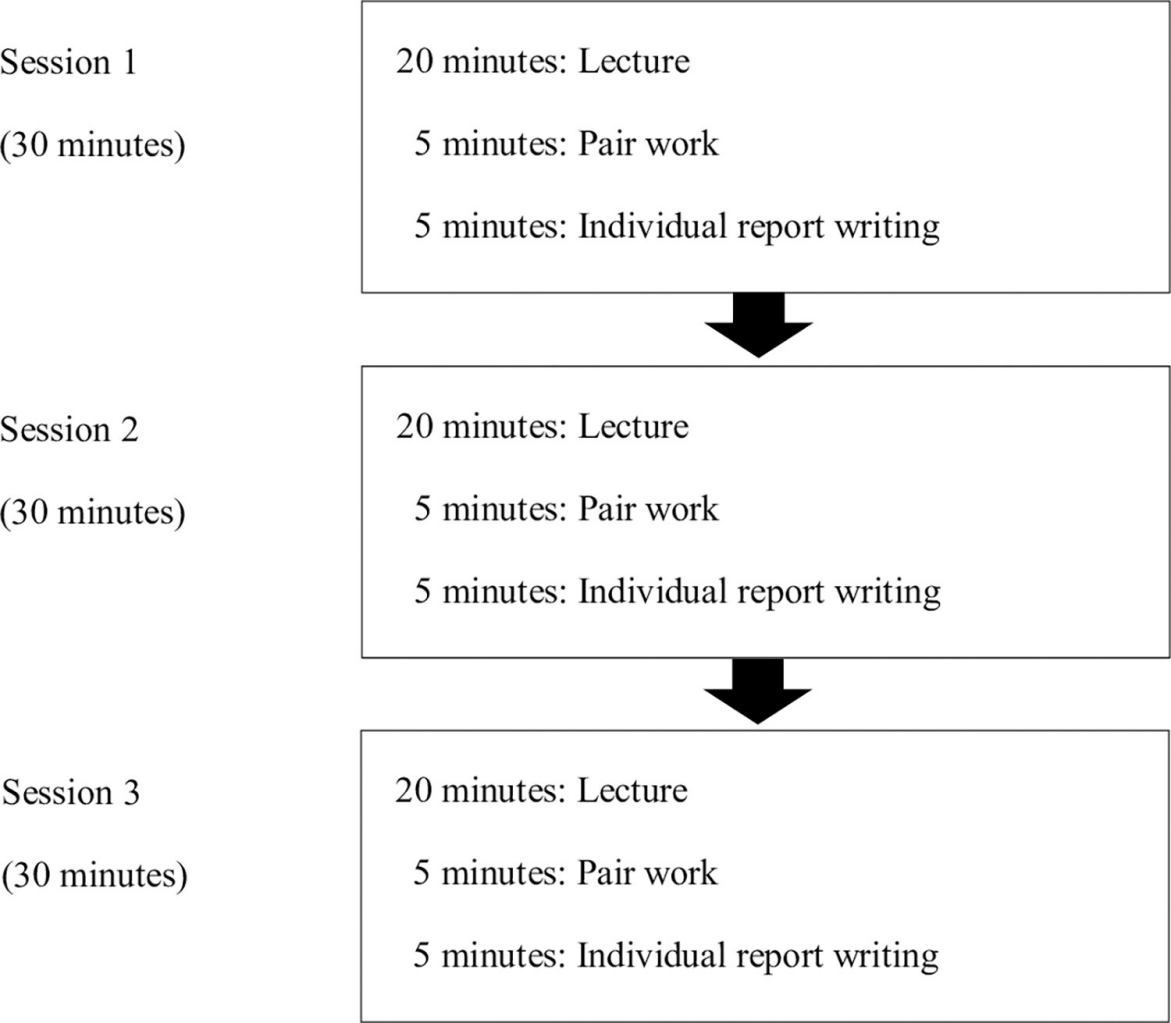
Flow chart of the experimental process.

The classroom practice in this study was conducted in the traditional “lecture” style with more than 100 students. This study was conducted in two classes, both of which were lecture courses in the field of psychology at universities. The content of the two lectures included an overview of “what is intelligence,” “development of new intelligence theories,” “intelligence and academic achievement,” and “metacognition and learning.” The 90-minute class was divided into three sessions, and 30 minutes per session was planned as the approximate time allocation. Each session consisted of 20 minutes of lectures, five minutes of pair work, and five minutes of individual report writing. At the beginning of the lecture, students were asked to pair up freely with classmates near their seats. During the pair work and collaborative learning, the roles of the speaker and listener were alternated halfway so that the interaction did not become one-sided. Themes for discussion of lecture content were presented to encourage dialogue in pairs. Subsequently, we introduced the theme of the report based on the dialogue that involved creative thinking. While writing the essay, the students were asked to quote the ideas expressed by their partners and describe them in an original way. The flow chart of the experimental process is shown in Fig 2.

## Results

### Evidence based on internal structure

Confirmatory factor analysis was conducted to examine the factorial validity of each scale. A four-factor structure for interpersonal motivation and three-factor structure for engagement were assumed and verified. The parameters were estimated using the maximum likelihood method. The goodness of fit was as follows: for interpersonal motivation, χ^2^(84) = 429.397, *p* = .000, χ^2^/*df* = 5.112, RMSEA = .108, 90% CI [.098, .118], SRMR = .089, CFI = .859, TLI = .823; and engagement, χ^2^(74) = 258.925, *p* = .000, χ^2^/*df* = 3.499, RMSEA = .084, 90% CI [.073, .096], SRMR = .041, CFI = .949, TLI = .937. The indicators of the two scales had satisfactory values. Factor loadings were higher than .537 for all scales.

Reliability coefficients were calculated for the four types of interpersonal motivation and three aspects of engagement. One external regulation item was deleted because of its low reliability. As shown in Table 1, these values were approximately satisfactory. There were three sessions in this class and each student wrote three reports. The reliability coefficients were calculated based on three reports, and the values were approximately satisfactory.

**Table 1:** Reliability and validity analysis of interpersonal motivation, engagement, and performance.

	*n*	*Mean*	*SD*	*α*	*ω*	*CR*	*AVE*
External Regulation	348	1.99	0.81	.66	.67	.66	.39
Introjected Regulation	347	2.90	0.83	.70	.73	.71	.40
Identified Regulation	349	3.64	0.83	.79	.80	.79	.49
Intrinsic Motivation	349	3.89	0.92	.90	.92	.90	.70
Behavioral Engagement	349	5.26	1.08	.92	.94	.92	.70
Emotional Engagement	347	4.71	1.31	.92	.93	.92	.69
Cognitive Engagement	347	4.79	1.05	.83	.87	.83	.54
Performance	352	2.10	0.49	.72	.73		

Next, the composite reliability (CR) values were calculated, which are shown in Table 1. These values were approximately satisfactory. In addition, to examine the convergent validity of the scales, average variance extracted (AVE) values were also calculated. As shown in Table 1, these values were approximately satisfactory.

Following prior studies, the mean values of the items were calculated for each subscale of interpersonal motivation and engagement, and the scale scores were used in the following analyses. The average score of the three reports was calculated and used as an index of performance. The means and *SD*s of each scale score are presented in Table 1.

### Evidence based on relations between conceptually related variables

To examine the construct discriminant validity of the scales, the AVE square root of each construct was calculated. As shown in Table 2, the results were approximately satisfactory.

**Table 2:** Results of correlation analysis between interpersonal motivation, engagement, and performance.

		1		2		3		4		5		6		7
1	External Regulation	.*63*												
2	Introjected Regulation	.46	***	.*63*										
3	Identified Regulation	-.16	**	.17	**	.*70*								
4	Intrinsic Motivation	-.28	***	-.01		.78	***	.*84*						
5	Behavioral Engagement	-.12	*	.09		.34	***	.32	***	.*84*				
6	Emotional Engagement	-.11	*	.06		.45	***	.46	***	.67	***	.*83*		
7	Cognitive Engagement	-.02		.08		.36	***	.33	***	.66	***	.61	***	.*73*
8	Performance	.03		.07		.11	*	.06		.18	***	.11	*	.09

* *p* < .05.

** *p* < .01.

*** *p* < .001.

Note: Italicized numbers represent the AVE square root of each construct.

The results of calculating the correlation coefficients between interpersonal motivation, engagement, and performance are presented in Table 2. A simplex structure was identified among the four kinds of interpersonal motivation, as assumed by self-determination theory. In a continuous sequence from external to internal regulation, adjacent variables showed significant positive correlations (e.g., identified regulation and intrinsic motivation). There was also a significant negative correlation between distantly located variables (e.g., external regulation and intrinsic motivation). There were also significant weak to moderate positive correlations among all three aspects of engagement.

Regarding the correlation between interpersonal motivation and engagement, there was a rather weak but significant negative correlation between external regulation and behavioral and emotional engagement. There was a significantly weak positive correlation between the identified regulation and the three aspects of engagement. In addition, there was a significantly weak positive correlation between intrinsic motivation and the three aspects of engagement. Finally, for performance, there was a fairly weak but significant positive correlation with identified regulation, behavioral engagement, and emotional engagement.

### Analysis of path model

**Fig 3:**
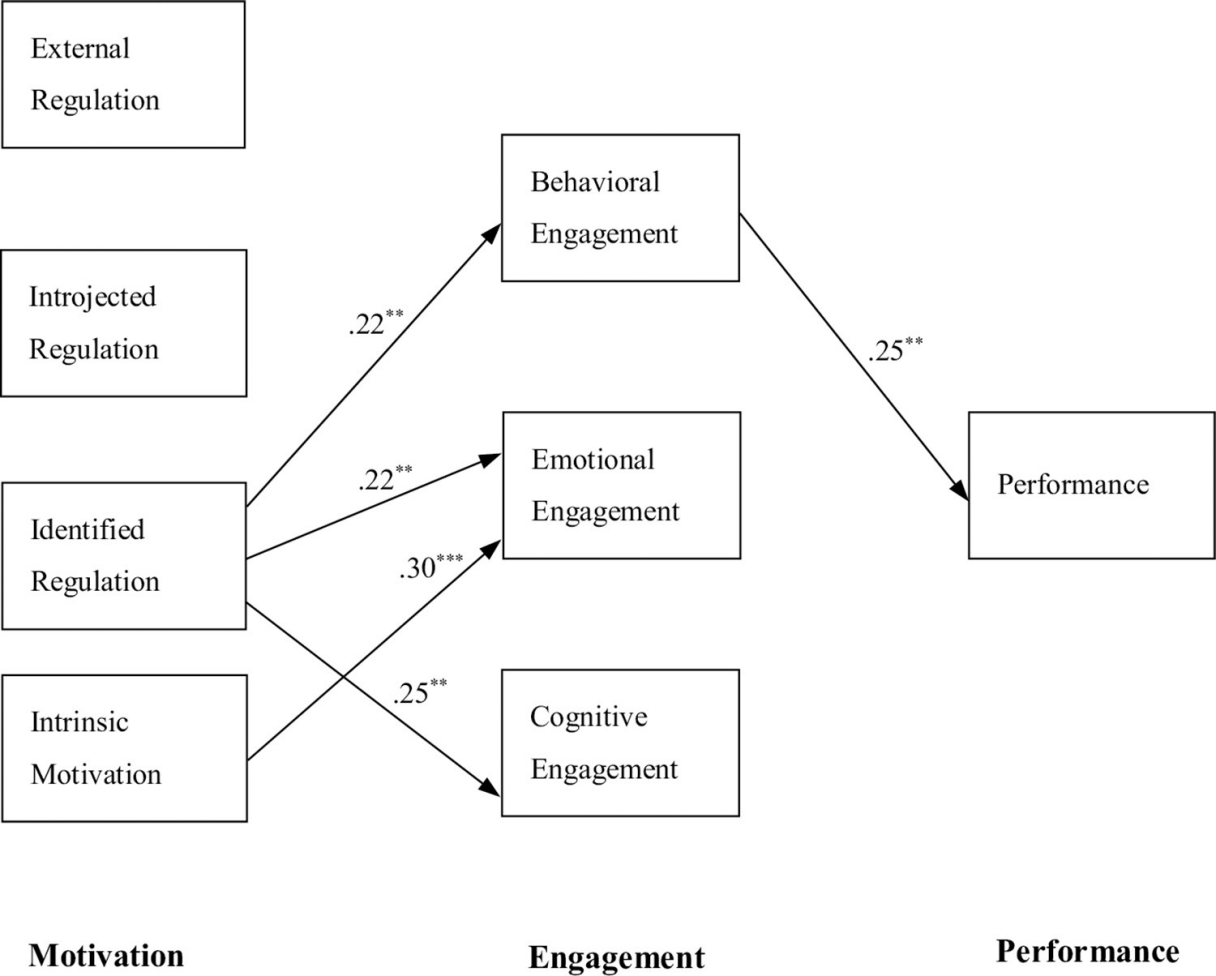
Result of structural equation modeling analysis on the path model for the interpersonal motivation, engagement, and academic performance. ** *p* < .01. *** *p* < .001. Note: Statistically significant paths are shown as solid lines with path-coefficient estimates.

We hypothesized a path model with interpersonal motivation as the antecedent, through engagement, and ultimately to determine performance, which was verified by structural equation modeling. Missing values were processed using the full information maximum likelihood method. The fit indices of the model were satisfactory, with χ^2^(18) = 20.139, *p* = .325, χ^2^/*df* = 1.119, RMSEA = .018, 90% CI [.000, .052], SRMR = .028, CFI = .998, and TLI = .996. The results of these analyses are presented in Table 3 and Fig 3.

**Table 3:** Results of structural equation modeling analysis on the path model for interpersonal motivation, engagement, and academic performance.

Criterion		*B*	95% CI	*SE*	*β*		*p*
	Predictor						
Behavioral Engagement							
	External Regulation	-.13	[-.28, .03]	.08	-.10		.12
	Introjected Regulation	.13	[-.02, .29]	.08	.10		.09
	Identified Regulation	.29	[.07, .51]	.11	.22	**	.01
	Intrinsic Motivation	.13	[-.08, .33]	.10	.11		.22
	*R* ^*2*^	.13					
Emotional Engagement							
	External Regulation	.03	[-.15, .21]	.09	.02		.77
	Introjected Regulation	.02	[-.15, .20]	.09	.01		.81
	Identified Regulation	.35	[.10, .60]	.13	.22	**	.01
	Intrinsic Motivation	.43	[.20, .67]	.12	.30	***	.00
	*R* ^*2*^	.24					
Cognitive Engagement							
	External Regulation	.08	[-.07, .23]	.08	.06		.31
	Introjected Regulation	.03	[-.12, .18]	.08	.02		.72
	Identified Regulation	.32	[.10, .53]	.11	.25	**	.00
	Intrinsic Motivation	.16	[-.03, .36]	.10	.14		.11
	*R* ^*2*^	.14					
Performance							
	Behavioral Engagement	.10	[.03, .17]	.04	.25	**	.01
	Emotional Engagement	.00	[-.06, .05]	.03	-.01		.89
	Cognitive Engagement	-.02	[-.09, .05]	.04	-.04		.62
	*R* ^*2*^	.05					

** *p* < .01.

*** *p* < .001.

Following the path flow, the statistically significant paths, in order of causality, were as follows. First, the weak positive paths from identified regulation toward all three aspects of engagement were substantial. Second, a weak positive path from intrinsic motivation was significant for emotional engagement. Finally, for performance, the weak positive path from behavioral engagement was statistically significant.

## Discussion

### Examining path models

The assumed causal model was verified using structural equation modeling. Based on the order of causal direction, the paths that were significant are discussed in turn.

As predicted, there were significant positive paths from identified regulation to behavioral, emotional, and cognitive engagement. Previous research [12, 13] has shown that identified regulation has a positive influence on academic achievement and school adjustment, which is consistent with the results of this study. In the first study to assess interpersonal motivation for collaborative learning, the role of identified regulation was shown to be especially important. This is a novel finding. Future research should include interpersonal motivation as an important antecedent factor in elucidating the learning process in classes that include collaborative learning.

By contrast, intrinsic motivation showed a significant positive path only for emotional engagement. Emotional engagement includes interest and enjoyment and intrinsic motivation consists of similar emotions. Intrinsic motivation was assessed before the lecture and was similar to the trait construct. Emotional engagement assesses the psychological state of the students while they are engaged in the class and is a construct that is more likely to change depending on the situation. Intrinsic motivation, a relatively stable antecedent, positively predicted emotional engagement, which is a situational factor. Behavioral and cognitive engagement were not related to intrinsic motivation, but this may be due to differences in psychological aspects. The scale items for intrinsic motivation used in this study were limited to questions examining emotional (e.g., fun, pleasant, delightful) rather than behavioral or cognitive aspects. Further verification is required in the future.

As hypothesized, a positive association was found between behavioral engagement and performance. This finding is consistent with previous reports [27–29]. Semester grades and final exam scores have often been used as indicators of performance, but this study revealed that performance was predicted by an index of creative and critical thinking writing based on classroom dialogues.

All path coefficients were found to be positive and significant, but the values were generally small. Possible reasons for this result are as follows: first, the scale of interpersonal motivation measured the tendency to be motivated in daily collaborative learning situations. The three dimensions of engagement were measured immediately after the lecture, in the context of the current situation; therefore, different levels of measurement could have weakened the association between these variables. Second, engagement was a measure based on self-evaluation, whereas others’ evaluations were used to measure performance. These differences in evaluators may have weakened the association between engagement and performance. Essentially, there may be some difficulty for engagement to produce educational outcomes in collaborative learning situations that require interaction with others. In particular, the performance tasks in this study required deep thinking and high creativity through dialogue with a classmate, which may not have been easy.

### Implications for educational practice and limitations of this study

As the hypothesized causal model was verified, the following practical suggestions were considered. First, university educators need to support identified regulation among other interpersonal motivations. It is important to encourage university students to realize the value and importance of interacting with others through cooperative learning, and to provide them with such experiences. Once students identify with the value of interpersonal relationships, identified regulation leads to behavioral, emotional, and cognitive engagement, which in turn leads to active participation in university classes, including collaboration. Focused attention and persistence in deep thinking through dialogue with classmates results in superior performance.

In addition, university educators should help students find fun and excitement in interacting with others through collaborative learning. If students are intrinsically highly motivated beforehand, they can enjoy and become interested in university classes that involve collaboration. As there were high positive correlations between engagement in the three behavioral, emotional, and cognitive dimensions, there may be potential for changes in engagement in other aspects as well.

Although the internal correlations of engagement are significant, it is necessary to keep in mind that behavioral engagement is particularly important in relation to performance and learning outcomes. Instructors are required to create a learning environment that allows students to focus on and sustain their attention and actively participate in collaborative learning. Following the path model, the necessary practices can be summarized as follows. First, educators should encourage students’ identified regulation and intrinsic motivation. In the classroom, students should create a learning environment that allows them to engage behaviorally. As this psychological learning process unfolds, students can think and learn deeply and creatively based on their dialogue with their classmates.

In recent years, research on teaching and learning has focused on self-regulated [35–38] and socially shared regulation of learning [39–42]. While it is important for students to learn by self-regulating their own cognitive and motivational processes, it is also necessary for them to learn by regulating each other’s cognition and motivation through collaboration and dialogue. Interpersonal motivation and engagement in collaborative learning should be deeply related to socially shared regulation of learning, and future research is required to examine this in more detail.

The limitations of this study are as follows. First, the study was conducted at a single university, which decreases the likelihood that findings are generalizable. Second, the assessment of engagement in this study examined active participation in classroom situations, including collaborative learning. Two aspects of engagement must be distinguished and demonstrated: engagement in collaboration with classmates and engagement in personal thinking and writing activities. Third, in this study, collaboration was through dialogue between pairs. However, there can be a variety of teaching styles and techniques that promote cooperative learning [30, 33, 43]. It is necessary to further verify the results by changing the cooperative learning method, group size, and task content.

## Conclusions

Using structural equation modeling, this study validated a path model from interpersonal motivation through engagement to superior performance. We were able to apply interpersonal motivation to collaborative learning situations and clarify the process of its influence. This is the novelty and significance of this study, and provides a possibility for future research. From the perspective of motivation, this study suggests a new direction for university classes and learning processes.

## Supporting information

S1 Appendix Questionnaire items. (DOCX)

S1 File Data on each measure. (XLSX)
