# Supplementary material for: Examining the causal relationships between interpersonal motivation, engagement, and academic performance among university students
Source: PLoS One. 2022 Sep 15;17(9):e0274229. doi: 10.1371/journal.pone.0274229 (PMC9477296; doi:10.1371/journal.pone.0274229)
Supplement: S1 Appendix — (DOCX) [file pone.0274229.s001.docx]

**Appendix**

Questionnaire items to assess interpersonal motivation and engagement are listed below.

**External regulation**

Because I am asked by those around me to build relationships with my members

Because the group members will get upset if I don’t stay with the members

Because the group members will be disappointed if I am not close to them

Because the group members will talk to me^*1^

Note: ^*1^ indicates item removed from the current analysis.

**Introjected regulation**

Because I feel anxious if I am not close to group members

Because if I don’t build a relationship with the group members, I will be in trouble later

Because it is embarrassing not to be able to form relationships with group members

Because I should be close to my group members

**Identified regulation**

Because it is important for me to spend time with group members

Because relationships with members are meaningful to me

Because being with the group members makes me happy

Because it is valuable to get to know the members of my group well

**Intrinsic motivation**

Because it is fun to spend time with group members

Because it is interesting to talk with group members

Because being with group members makes the pleasant time longer

Because it is delightful to get to know the members of the group

**Behavioral engagement**

I paid attention in pair work

I tried very hard in pair work

I worked on the pair work without giving up until the end

I worked energetically on pair work

I was enthusiastic about the pair work

**Emotional engagement**

When we worked on something in pair work, I felt interested

Pair work was fun

I like pair work

I found the pair work amusing

I was excited when we were doing the pair work

**Cognitive engagement**

I devised the way to promote learning through pair work

I devised the way to deepen the ideas in the discussion through pair work

I worked on it, thinking about whether there was an efficient way to proceed

I worked on it, trying to figure out how to make the discussion better
